# The link between endocrine parameters, serum lithium concentrations, and cognitive functions among individuals with affective disorders at risk of suicidal behavior. A study protocol

**DOI:** 10.1371/journal.pone.0311347

**Published:** 2024-12-16

**Authors:** Vilma Jakiene, Gil Zalsman, Julius Burkauskas, Virginija Adomaitiene, Eimantas Zauka, Rima Naginiene, Narseta Mickuviene, Vesta Steibliene

**Affiliations:** 1 Laboratory of Behavioral Medicine, Neuroscience Institute, Lithuanian University of Health Sciences, Palanga, Lithuania; 2 Geha Mental Health Center, Clalit HMO, Faculty of Medicine, Tel Aviv University, Tel Aviv, Israel; 3 Division of Molecular Imaging and Neuropathology, Department of Psychiatry, Columbia University, New York, New York, United States of America; 4 Clinic of Psychiatry, Lithuanian University of Health Sciences, Kaunas, Lithuania; 5 Laboratory of Toxicology, Neuroscience Institute, Lithuanian University of Health Sciences, Kaunas, Lithuania; Duke University School of Medicine, UNITED STATES OF AMERICA

## Abstract

**Introduction:**

Suicidal behavior is prevalent among individuals with mental disorders and constitutes a major global, clinical, and public health concern. It is generally accepted that the majority of persons who die by suicide are suffering from psychiatric disorders in most cases and that affective disorders make up the single commonest group. Suicide risk is highest during the years immediately following the onset of affective disorders; therefore, identifying risk factors and successful treatment of initially non-suicidal individuals with affective disorders can prevent developing suicidal behavior, help detecting, and predict it. Clarifying risk factors for individual types of major affective disorders could enhance earlier identification of suicidal risk, support preventive interventions and improve the treatment and prognosis of individuals at risk. We have developed a study protocol with the aim to address the complex interaction of endocrine parameters, lithium trace concentrations and cognitive functions with affective disorders and risk of suicidal behavior.

**Method:**

A cross-sectional study will be done among individuals hospitalized after a suicidal attempt for treatment of affective disorders (group 1), hospitalized for the treatment of affective disorders without life time history of suicidal behavior (group 2), and control group individuals without life time history of mental disorders or suicidal behavior (group 3). Based on this study design we calculated the appropriate study sample size (N = 210). Venous blood samples of study participants will be collected for the evaluation of endocrine parameters, serum lithium concentrations, liver and kidney function. Data on sociodemographic factors, cognitive functions, history of mental disorders, and suicidality risk will be evaluated using validated questionnaires and instruments. Associations of endocrine parameters, serum lithium concentrations, cognitive functions, and suicidality risk will be analyzed using descriptive and inferential statistics, including linear and logistic regression. Based on this study design we calculated the appropriate study sample size (N = 210). Power analysis has shown that this sample size is appropriate for detection of significant differences between the groups.

**Conclusion:**

The findings of the potential influence of the associations between serum lithium trace concentrations, endocrine parameters, and cognitive functions on suicidality risk in individuals with affective disorders may help clinicians effectively plan suicide prevention and timely implement actions for treatment.

## Introduction

A number of neurobiological models [1–3] and biomarkers [4–7] have been proposed to explain suicidal behavior. However, among individuals with affective disorders, it is difficult to predict suicidal behavior due to a low base rate of cases, even in high-risk groups and given the multi-causal nature of suicidal behavior [8, 9]. Neuroimaging, genetic, and neuroendocrine findings support the existence of biological basis related to suicidal behavior and the need to identify acute, modifiable, and treatable clinical biomarkers that would provide substantial benefits in multidimensional suicide preventive strategies and treatment planning [10, 11].

Increased risk of suicidal behavior is known to be associated with abnormalities in the serotonergic system, hypothalamic–pituitary–adrenal (HPA) axis, lipid metabolism, immune system, and neuronal plasticity [12]. Structural brain changes are found in individuals who attempted suicide and in individuals with mental disorders [13–15]. These brain changes could lead to changes of cognitive [16] functions and personality traits, such as impulsivity and cognitive rigidity, are strongly associated with risky behavior and suicidality [17–19]. It has been observed that cognitive impairment is associated with depressive disorder and higher risk of suicide [20]; while worse cognitive functioning is associated with more frequent suicidal ideation in individuals with depression [21]. However, most of these studies were based on small or non-representative samples. Some studies reported low plasma testosterone levels after suicide attempts [22–24]. Testosterone and other androgens might have antidepressant properties, and might be involved in the pathophysiology of mood disorders and suicidal behavior. Sher et al. inferred that testosterone may be a contributing factor for the pathophysiology of suicidal behavior due to its direct effects on mood, cognition, and aggression [25]. The relationship between low serotonergic function and suicidal behavior is also indicated by a blunted prolactin response to serotonin that is released by fenfluramine in suicide attempters with major depression or personality disorders compared to controls [26, 27].

Thyroid dysfunction is a very common condition that influences the entire human body, including cognitive function [28] and mental health [29], and is associated with multiple neuropsychiatric conditions, such as affective disorders and suicidal behavior [30–32]. The prospective study, performed by Sliuoziene et al. found that individuals with euthyroidism and with high suicide risk had lower mean free triiodothyronine (FT3) concentrations and lower mean free thyroxine (FT4) concentrations [33]. Pompili et al. found similar differences in FT3 concentrations; they indicated that suicide attempters were 2.27 times less likely to have higher FT3 values than non-attempters [34]. However, reports on the relationship between thyroid axis function and suicidality are scarce and have significant heterogeneity. Therefore, more importantly to understand how thyroid dysfunction may influence the development and interaction on suicidality risk with mood disorders and cognitive changes.

A negative relationship between severity of depressive symptoms and thyroid-stimulating hormone (TSH) concentrations among suicidal individuals showed that TSH is more related to affective symptomatology than to suicidal behavior. Thyroid hormones play an important role in regulating mood and emotional balance through their impact on the serotonin and noradrenaline systems; while disruptions in the balance of these hormones can contribute to affective symptomatology and increase of suicidality risk [35, 36]. Earlier studies showed an association of thyroid dysfunctions and cognitive changes, mood symptoms and suicidal behavior [32, 37–39]. Researchers found that thyroid dysfunction affect impaired memory, psychomotor slowing, and severity of depression, mood instability, and aggression impulsivity. In addition, low FT3 syndrome is known as a predictor of poor outcomes in patients with severe somatic illnesses and mental disorders. A few studies have provided evidence of the relationship between lower FT3 and worse psychomotor speed, and association of higher levels of FT4 with poorer cognitive outcomes [40–42].

Almost every metabolic process operates by involving trace elements. Therefore, biochemical and metabolic interactions, very relevant to human medicine as well as in diagnostics and therapy. There is data on the relation between trace elements concentration in brain cortex and suicidal behaviors [43]. Several studies have investigated the effects of micro-dose lithium on mood among individuals and found that higher serum lithium concentrations were associated with a lower incidence of suicide attempts [44–46]. Schoepfer et al. (2021) showed significantly higher endogenous lithium concentrations in white matter compared to grey matter as a general trend in individuals without a history of suicidal behavior and lower lithium concentrations in emotion-modulating regions in individuals with a previous suicide attempt [47]. There is evidence that lithium may have neuroprotective effects, preserving the function of neurons and neuronal circuits [48]. It is also postulated that lithium may exert its ‘anti-suicidal effects’ by lowering testosterone levels [49]. Lithium, a trace element present in small quantities within living organisms, primarily serves the function of supporting thyroid activity [50]. Additionally, it plays a role in managing affective symptoms [51, 52], while also exerting anti-inflammatory and antioxidant effects in regulating nervous system metabolism [53]. Lithium is normally obtained through food. According to estimates, cereal grains and vegetables can provide 66–90% of the daily lithium consumed. The remaining required daily norm of lithium is supplied by animal-derived foods and drinking water [54]. Enderle et al. (2020) found people who consumed more fruits, vegetables, and tea had higher lithium concentrations [55]. Although this trace element is not officially considered a micronutrient, some authors have suggested its provisional recommended intake set at 1,000 μg/day for a 70 kg adult to maintain patients’ physiological levels [51, 54, 56] and prevent mood alterations [57]. Other minerals such as selenium (Se), zinc (Zn), and iodine (I) are also important to thyroid metabolism and function, and correlate with thyroid autoimmunity [58]. However, excess or deficiency of these trace elements may be disturbed abnormal thyroid function and may cause oxidative stress and neurologic disturbances, contributing to mental disorders [59] or suicidal behavior [60]. Moreover, lithium plays a role in managing affective symptoms [51, 52], while also exerting anti-inflammatory and antioxidant effects in regulating nervous system metabolism [53].

### The aims

*The primary aim* of the present study is to determine the serum lithium trace concentrations in individuals with affective disorders and assess their association with suicidal behavior.

The secondary objectives:

1. to investigate associations between serum lithium concentrations and endocrine parameters in individuals with affective disorders, with and without a history of suicidal behavior, and healthy controls;
2. to provide a model for understanding the interactions between serum lithium concentrations, endocrine parameters, cognitive functions, and the risk of suicidal behavior in individuals with affective disorders.

Statements of the hypotheses.

We hypothesized, that:

1. Lower lithium trace concentration in serum is associated with higher risk of suicidal behavior among individuals with affective disorders.
2. Individuals with affective disorders and with a history of suicidal behavior have significantly lower normal serum levels of thyroid-stimulating hormone (TSH) and differ in free-triiodothyronine (FT3) concentrations when compared to controls.
3. Prolactin and free-testosterone serum concentrations differ among individuals with affective disorders and with a history of suicidal behavior in comparison to individuals without a history of suicidal behavior, and may mediate the effect of suicidal behavior.
4. Interactions between lower serum concentrations of trace lithium, reduced levels of thyroid axis hormones (TSH and FT3), lower prolactin and free testosterone concentrations, and cognitive deficits in the executive function domain may mediate the effects on suicidal behavior in individuals with affective disorders, independent of clinical risk factors, depressive and anxiety symptoms.

## Method

### Study design and participants

**Fig 1:**
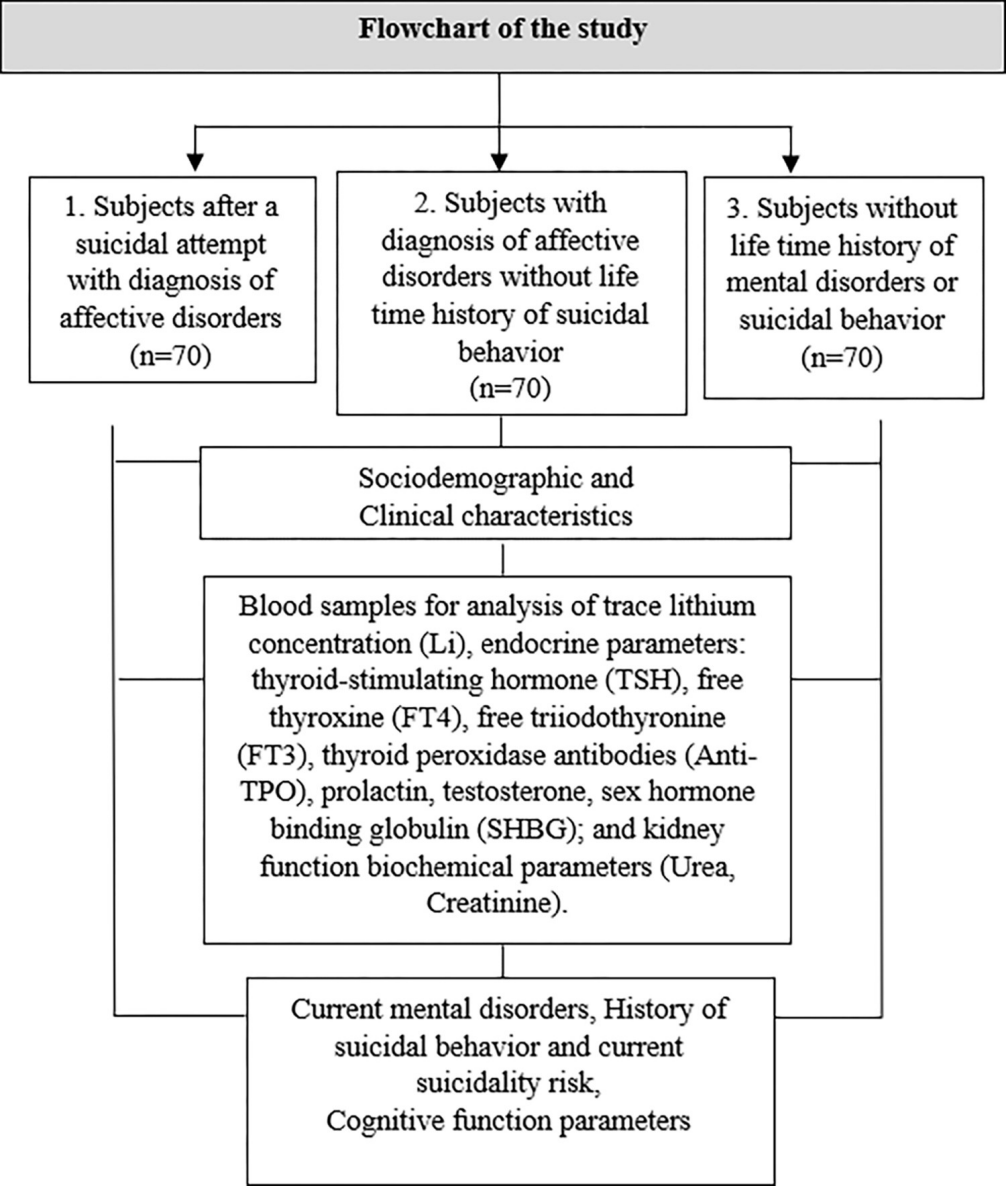
Study design overview.

A cross-sectional controlled study design will be used. All individuals, consecutively hospitalized at the Psychiatric Department of Hospital of the Lithuanian University of Health Sciences after a suicidal attempt (SA) for the treatment of affective disorders (group 1), consecutively hospitalized at the Stress-related Disorders Department of the Lithuanian University of Health Sciences Neuroscience Institute, Palanga Clinic for the treatment of affective disorders without life time history of suicidal behavior (group 2), and individuals without life time history of mental disorders or suicidal behavior admitted to the Outpatient Department of the Lithuanian University of Health Sciences Neuroscience Institute Palanga Clinic for prophylactic evaluations or for physical rehabilitation (group 3), will be invited to participate in this study (Fig 1).

*Inclusion criteria* for the whole study population are: 18 years of age and older; ability to understand the essence of the research and sign the informed consent form; understands, speaks, and writes in Lithuanian; and for the study group 1: patients diagnosed with affective disorders and hospitalized to an acute psychiatric department after an suicide attempt (SA); and for the study group 2: patients with affective disorders without a life-time history of SA admitted for treatment at a stress-related disorders unit.

*Exclusion criteria* for the study groups 1 and 2 are: organic mental disorders, schizophrenia, diagnosis of mental retardation, unstable somatic/neurological state, thyroid disorders or using thyroid medications, and using medication that contain lithium, pregnant or breastfeeding; and for the individuals of group 3: any life time of mental disorder or mental and behavioral disorder due to the use of psychoactive substances (except tobacco), life time history of suicidal behavior, using medication that contains lithium, thyroid disorders or using thyroid medications; pregnant or breastfeeding.

A total of 210 subjects are expected to participate in this study. The study sample was calculated based on standard sample size formulas [61], selecting 5% error and 95% probability (unilateral 5% test and 80% power) to present statistically significant conclusions.

Consistent with ethical guidelines, participants will have the right to opt out of the study at any time if they wish. If participants withdraw their consent or if the research team learns that a participant does not meet the inclusion or exclusion criteria during the study, data collection will be stopped and all collected biological material and data will be destroyed.

### Measurements

This study will use a set of validated instruments described in Table 1. The personal data of all participants will be treated anonymously and will be assigned with an individual code. A series of steps and measures will be implemented in the study, as outlined in Table 1.

**Table 1:** Steps and measurements in the study.

Steps	Variables	Instruments	Measurements
1	Blood samples	The venous blood samples will be collected from fasting subject (amount: 8 mL). The blood serum will be separated by centrifugation at 3000 × g and then frozen at –70°C. The biochemical parameters will be analyzed using electrochemiluminescence immunoassay (Advia Centaur XP 2016; Siemens Osakeyhtio, Espoo, Finland). Blood serum analyses will be performed in a certified biochemical laboratory.	Blood samples (serum) will be analyzed for concentrations of TSH (mIU/L) norm (0.55–4.78); FT4 (pmol/L), norm (11.5–22.7); FT3 (pmol/L), norm (3.5–6.5); thyroid peroxidase antibodies (Anti-TPO, U/mL), norm (<60), prolactin (mIU/L), norm (male: 0–424, female: 0–530), testosterone (nmol/L), norm (male: 10–35, female: 0.5–2.4). SHBG (nmol/l), norm (12.5–6.6) and trace elements of lithium (Li, μg/L), selenium (Se, μg/dL), zinc (Zn, μg/dL), and iodine (I, μg/dL). Kidney function biochemical parameters (Urea, Creatinine) will be used for evaluation of thyroid hormones and Li metabolism.
2	Sociodemographic characteristics and clinical history	Questionnaire	Age, gender, marital status, education level, living area, socioeconomic status, medical history, medication and food supplement consumption habits, nutrition, smoking, and alcohol use habits.
3	Mental health outcomes	Diagnostic Statistical Manual of Mental Disorders (DSM-5)	Subjects will be assessed and diagnosed with a mental disorder by a psychiatrist using the Diagnostic Statistical Manual of Mental Disorders (DSM-5).
4	History of suicidal behavior and current suicidality risk	Mini International Neuropsychiatric Interview (M.I.N.I. 7.0.2)	Psychiatric diagnoses, including an evaluation of past and current suicidality, was performed using the Mini International Neuropsychiatric Interview.
5	Cognitive function	Cambridge Neuropsychological Test Automated Battery (CANTAB)	(CANTAB) will be used to evaluate alterations in executive function Specifically, the following tests will be used: • Motor screening Task (MOT)–sensorimotor function. • Cambridge Gambling Test–decision making (impulsivity, risk taking) • Delayed Matching to Sample–short-term visual recognition memory; • Intra-Extra Dimensional Set Shift (IED)–set-shifting, mental flexibility; • Match to Sample Visual Search (MTS)–attention and visual searching; • One Touch Stockings (OTS)–spatial planning and the working memory; • Rapid Visual Information Processing (RVP)–sustained attention; • Spatial Working Memory (SWM)–working memory.

All subjects will be assessed and diagnosed with a mental disorder by a psychiatrist using the Diagnostic Statistical Manual of Mental Disorders (DSM-5) [62]. Psychiatric diagnoses, including an evaluation of past and current suicidality will be evaluated using the Mini International Neuropsychiatric Interview (M.I.N.I.7.0.2) [63]. Suicidality risk was assessed using Section B of the MINI. Based on the sum of the weighted scores of the “yes” items, a total of 1–8 points were classified as ‘low-risk’, 9–16 points as ‘moderate-risk’, and 17 points and higher as ‘high-suicidality risk’. Cambridge Neuropsychological Test Automated Battery (CANTAB) will be used to evaluate alterations in executive function [64]. CANTAB tests have been validated in behavioral and psychopharmacological studies on healthy volunteers and in a range of patient groups [65].

### Statistical analysis

All statistical analyses of the collected data will be conducted using the Statistical Package for the Social Sciences software v.29.0.0.0 (IBM SPSS, Chicago, IL, USA).

Prior to statistical analysis, all data will be reviewed for quality, distributions, and missing data bias (e.g., missing at random). The collected data will be analyzed using descriptive statistics. Mathematical transformations will be performed when necessary to normalize scores. The descriptive data will be presented as means (geometrical means, if necessary) and standard deviations alongside 95% confidence intervals or median (25th–75th percentile), and categorical variables as the frequency and percentage. A Shapiro–Wilk test and inspection of shape parameters such as skewness and kurtosis coefficients will be performed to check the normality assumption. Levene’s test will be used to test if samples have equal variance. Differences between two groups will be assessed by Student or Welch t tests, or Mann–Whitney U test, as appropriate. Comparison among multiple groups (3 groups) will be performed by one-way ANOVA. Inequality of variance will be tested by performing Welch t test. Bonferroni correction will be used for multiple testing (p = 0.05/3 = 0.0167). Multivariable linear and logistic regression models will be applied including suicidal behavior as a dependent variable and sociodemographic, endocrine parameters, concentrations of the trace element lithium, cognitive functions, and their interaction as covariates.

### Ethical consideration

This study has been approved by the Kaunas Regional Biomedical Research Ethics Committee (protocol No. BE-2-23, 17/02/2023). In the period from 01/03/2023 to 28/02/2025, all subjects will be included in the study, if they agree to participate and sign the informed consent form. The study will be conducted in accordance with Good Clinical Practice guidelines and the principles of the Declaration of Helsinki. The data will be processed in accordance with the Personal Data Processing Rules of the institution (LUHS), Regulation 2016/679 of the European Parliament and of the Council of 27 April 2016 on the protection of natural persons in the processing of personal data and on the free movement of such data, the Law on the Legal Protection of Personal Data of the Republic of Lithuania, and other legal acts regulating the processing of personal data. All subjects will be informed of the right to opt out of the study at any time. The survey data will be stored in archive files at the LUHS in a locked room for at least 5 years. Any information about the subjects collected during the study will be depersonalized, ensuring the subjects’ confidentiality, anonymity and data protection.

## Discussion

This cross-sectional controlled study will allow to determine serum lithium trace concentrations in individuals with affective disorders and to evaluate whether trace lithium concentration is associated with a history of suicidal behavior, current suicidality and to evaluate the interaction with different endocrine parameters and cognitive functioning. Moreover, this research project may provide an important information about the interactions of different biological factors for a better understanding of their contribution to the risk of suicidal behavior.

Due to the scarcity of evidence and the conflicting findings among observational studies, we aim to be the first ones to explore the association of the natural serum lithium concentration, thyroid axis hormones, prolactin, testosterone concentrations and cognitive functions in subjects with suicidal behavior and/or diagnosis of affective disorders. Therefore, the evaluation of the endocrine parameters, lithium trace concentrations, and cognitive functioning among individuals with and without affective disorders and suicidal behavior, and comparison to healthy control individuals, may help better understand the specific associations and complexity of suicidality risk factors.

A meta-analysis of ecological studies have suggested the protective effect of micro-dose lithium in drinking water against suicide [66, 67]. Some researchers have even theorized that adding lithium to drinking water could potentially reduce the rate of suicide among the general population; however, the association between body lithium level and suicidal behavior is unknown. In fact, lithium’s anti-aggressive and suicide-preventive capacity in clinical practice is well established [68].

Several studies have examined the effect of micro-dose lithium on mood [44, 51], cognitive impairment [69] and suggested an important role in suicidality [45, 47]. It is known, that lithium modulates the serotonergic and noradrenergic systems, which are closely linked to mood regulation and the pathophysiology of mood disorders. Moreover, lithium inhibits glycogen synthase kinase-3 (GSK-3), an enzyme involved in various cellular processes, including those that affect mood and cognition. However, when biological processes and intracellular pathways of lithium are disrupted, reduced efficiency of executive functions, mood dysregulated, and neurobiological factors cause changes in thinking and decision-making that increase the risk of suicidal behavior [70]. A recent study showed that the trace element lithium levels in the body were lower in suicides than in non-suicides, suggesting that even micro-dose lithium might have an important role in suicide [46]. However, the actual lithium concentration needed to induce anti-suicidal effects in people with affective disorders remains unclear; and little is still known about trace lithium distribution and any associated metabolic effects in the human body.

Although numerous studies have examined suicide risk processes associated with thyroid dysfunction, cognitive impairment and lithium treatment, there is a distinct paucity of research on patients with affective disorders, despite the fact that this population suffers a higher risk of suicide relative to the general population.

Our previous research on brain structural changes in subjects with suicidal behavior [15], thyroid axis functioning in suicidal individuals [71], and findings of the inverse relationship of lithium concentration in drinking water with suicide mortality rates [72, 73] encourages us to hypothesize about existence of a biological parameters, related not only to the affective disorders, but to the risk of suicidal behavior.

The evaluation of individuals with affective disorders, previous suicide attempts, and individuals who survived and were hospitalized after a suicide attempt forces us to search the links among known biochemical and genetic factors, involving a variety of systems: major neurotransmitters [4], the HPA axis, neuroinflammatory processes, lipids, and neuroplasticity [74]. These links could be hormones of the thyroid axis, part of the neuroendocrine system that mainly regulates the body’s metabolism, and could act as active brain neurotransmitters [75–78].

Lithium has been regarded as efficacious in affective disorder treatment. However, the role of lithium as a trace element in the development of affective disorders is still questionable because the individual serum lithium level (obtained by regular nutrition through drinking water and food) is about a thousand times less than that used in therapeutic dosages (600–1200 mg/d) [79, 80]. Nevertheless, certain ecological studies indicate its potential efficacy even at such a low concentration with ranges up to several mg Li+/d [54, 81–83].

To date, there are no clear pathophysiological links between thyroid function parameters, prolactin, testosterone concentrations, cognitive functioning, and suicidal behavior, and the data on the relationship between trace lithium concentrations and the risk of suicidal behavior are conflicting. In developing suicide prevention and treatment guidelines for clinicians, modern psychiatry requires a clearer understanding of suicide risk. There is also a need for more thorough assessment and careful stratification of suicide risk, especially among high suicidality risk groups, to enhance the planning of clinical interventions and treatments.

A detailed examination of the data can help identify potential association of endocrine parameters, lithium trace concentrations and cognitive function that might explain how these factors mediate and influence suicidal behavior. Understanding these mechanisms is essential for developing targeted interventions to different populations and settings. We expect that lower levels of certain hormones, lower lithium trace concentration and changes in executive functioning might play a significant role in suicidal behavior in people with affective disorders, beyond what is explained by clinical symptoms alone. If the hypotheses are confirmed, the findings could have significant implications for the treatment and prevention of suicidal behavior in individuals with affective disorders.

### Strengths and limitations

This study offers a unique way to understand mechanisms including biological markers that may contribute to suicidal behavior in individuals with affective disorders. The study could help to answer the question ‘are specific biomarkers linked to affective disorder, to cognitive functions, or only to suicidal behavior’, as a specific behavior model.

Although, this cross-sectional study will limit the testing of causal relationship but will help to provide a model for associations of trace serum lithium concentrations, endocrine parameters, cognitive functions, and risk of suicidality in individuals with affective disorders.

## Conclusion

Based on the existing literature on risk of suicidal behavior among individuals with affective disorders, this study aims to provide a comprehensive understanding of the associations of trace serum lithium concentrations, endocrine parameters, cognitive functions, and risk of suicidality in individuals with affective disorders; elucidate and understand the mechanisms including biological markers that may contribute to suicidal behavior. It is expected that these findings will offer valuable insights to support evidence-based decision-making for clinicians and health policy makers on suicide prevention.
